# ASO Author Reflections: The Landmark Series: Minimally Invasive Pancreatic Resection—Past, Present, and Future

**DOI:** 10.1245/s10434-025-17595-0

**Published:** 2025-06-02

**Authors:** Adrian Diaz, Sarah Hays, Melissa E. Hogg

**Affiliations:** 1 Department of Surgery, University of Chicago, Chicago, IL USA; 2 Center for Healthcare Outcomes and Policy, University of Michigan, Ann Arbor, MI USA; 3 Department of Surgery, NorthShore University Health System, Evanston, IL USA

## Abstract

Minimally invasive pancreatic resection (MIPR) has emerged as a safe and effective approach for select patients with pancreatic ductal adenocarcinoma (PDAC), particularly for distal pancreatectomy. Ongoing randomized trials such as DIPLOMA 2 × 2 and PORTAL will further clarify its role in pancreatoduodenectomy, especially with robotic assistance. However, widespread adoption depends not only on evidence but also on access to technology and structured training programs. Expanding dedicated training, simulation-based education, and institutional support will be essential to ensure safe implementation. At the same time, emerging technologies such as augmented reality and next-generation robotics may help lower technical thresholds, lower prices, and accelerate adoption. The continued convergence of high-quality evidence, advanced surgical tools, and equitable implementation strategies will be critical to making MIPR a broadly accessible standard for PDAC, improving outcomes without compromising oncologic rigor. Barriers will need to be overcome to continue growth, such as limited availability of robotic platforms, high costs, and disparities in care.

## Past

Pancreatic ductal adenocarcinoma (PDAC) surgery has traditionally been performed via open resection, which remains the only potentially curative intervention. However, this approach is associated with significant morbidity and prolonged recovery, factors that have hindered the timely delivery of adjuvant chemotherapy—an essential component of multimodal treatment supported by level one evidence. Historically, optimal surgical outcomes have been best at high-volume centers and by surgeons with substantial technical expertise. Consequently, early efforts in the 1990s to introduce laparoscopic techniques to pancreatic surgery were met with skepticism, owing to the procedure’s inherent technical challenges, including complex anastomoses and extended operative times. These concerns contributed to the delayed adoption of minimally invasive pancreatic resection (MIPR), which did not gain broader clinical traction until the 2000s.

## Present

Over the past decade, however, accumulating high-level evidence has established MIPR as a safe and effective alternative to open surgery when performed by experienced teams.^1^ In particular, minimally invasive distal pancreatectomy has moved beyond the phase of mere acceptance and should now be considered the standard of care for resectable tumors of the pancreatic body and tail without vascular involvement. Two randomized controlled trials (RCT) from Europe have demonstrated superior perioperative outcomes with laparoscopic distal pancreatectomy, and the recent international DIPLOMA trial confirmed oncologic equivalence in terms of R0 resection and lymph node yield, while also reducing blood loss, shortening hospital stay, and faster recovery for MIPR, including robotic approach.^2^ Despite these advantages, access remains a major limitation—more so for robotic than for laparoscopic techniques.

For pancreatoduodenectomy, randomized trials such as PADULAP and PLOT have shown similar complication rates and oncologic outcomes between laparoscopic and open approaches.^3^ Nonetheless, the LEOPARD-2 trial, which was terminated early owing to increased mortality in the laparoscopic arm, highlighted the critical importance of rigorous training, volume, and patient selection.^4^ Recently, additional RCTs from China and Europe (EUROPA) have begun to examine robotic versus open pancreatoduodenectomy, further expanding the evidence base. Collectively, these data underscore that the benefits of MIPR are contingent upon performance in high-volume centers with well-structured training programs and coordinated multidisciplinary perioperative care.

## Future

Ongoing multicenter RCTs, such as the DIPLOMA 2 × 2 and PORTAL trials, are poised to further elucidate the comparative efficacy of robotic versus open pancreatoduodenectomy and provide critical insights into long-term oncologic and functional outcomes in patients undergoing PDAC.^1^ These investigations will help refine surgical decision-making and establish evidence-based guidelines for minimally invasive approaches. However, the continued dissemination of MIPR—both nationally and internationally—hinges on more than evidence alone. A pivotal barrier remains regarding equitable access to advanced surgical technologies, particularly robotic platforms, which are often limited to tertiary care centers and subject to significant institutional investment. Beyond access, widespread adoption will also depend on the availability of structured, subspecialty-focused training programs in robotic pancreatic surgery. Ensuring that surgeons receive dedicated, high-fidelity training is essential for maintaining patient safety and optimizing outcomes, particularly in complex resections such as pancreatoduodenectomy.

Concurrently, the advent of next-generation robotic systems promises to further ease the learning curve through enhanced instrument articulation, improved ergonomics, and integrated intraoperative imaging capabilities. Additionally, developments in augmented reality and artificial intelligence are showing early promise in enhancing intraoperative margin visualization, tissue imaging, and reducing operative times—innovations that are likely to integrate seamlessly into future MIPR workflows.^5^ Looking forward, the confluence of rigorous clinical trials, digital augmentation, and refined surgical education will be instrumental in driving MIPR toward routine application across a broader PDAC population. Such progress has the potential to optimize recovery trajectories without compromising oncologic standards, thus achieving a more patient-centered and efficient paradigm for pancreatic cancer surgery.

## Conclusion

Minimally invasive pancreatic resection (MIPR) represents a transformative evolution in the surgical management of pancreatic ductal adenocarcinoma (PDAC), offering the potential to improve perioperative outcomes while preserving oncologic integrity. Decades of technical refinement and growing high-level evidence now support MIPR, particularly for distal pancreatectomy. Nonetheless, the future of MIPR cannot rely on clinical data alone. Real-world implementation will demand pragmatic strategies to address access disparities, in terms of both physical infrastructure and workforce readiness.

Equally critical is the expansion of specialized training pathways that embed robotic proficiency into general and hepatopancreatobiliary (HPB) surgery curricula. Simulation-based education, case-volume thresholds, and credentialing standards will be essential for maintaining quality while expanding the procedural footprint. Surgical networks and remote mentoring technologies may be helpful for dissemination. Realizing this potential will require ongoing collaboration among surgeons, institutions, industry stakeholders, and societies to ensure that excellence in pancreatic surgery is a standard for all.
